# Real-life drug retention rate and safety of rituximab when treating rheumatic diseases: a single-centre Swiss retrospective cohort study

**DOI:** 10.1186/s13075-023-03076-w

**Published:** 2023-06-01

**Authors:** Alexandre Dumusc, Fahad Alromaih, Matthieu Perreau, Thomas Hügle, Pascal Zufferey, Diana Dan

**Affiliations:** 1 Department of Rheumatology, Lausanne University Hospital, 1005 Lausanne, Switzerland; 2 Faculty of Biology and Medicine, University of Lausanne, 1005 Lausanne, Switzerland; 3 Division of Immunology and Allergy, Lausanne University Hospital, 1005 Lausanne, Switzerland

**Keywords:** Rituximab, Off-label, Auto-immune diseases, Drug retention rate, Rheumatoid arthritis, Connective tissue disease, Vasculitis

## Abstract

**Background:**

In Switzerland, rituximab (RTX) is licenced for the treatment of rheumatoid arthritis (RA) and ANCA-associated vasculitis (AAV) but is frequently used off-label to treat other auto-immune diseases (AID), especially connective tissue diseases (CTD). We aimed to characterise the use of RTX in AID in a real-life Swiss setting and compare RTX retention rates and safety outcomes between patients treated for RA, CTD and AAV.

**Methods:**

A retrospective cohort study of patients who started RTX in the Rheumatology Department for RA or AID. The RTX retention rate was analysed using Kaplan–Meier survival curves. Occurrences of serious adverse events (SAE), low IgG levels and anti-drug antibodies (ADA) were reported.

**Results:**

Two hundred three patients were treated with RTX: 51.7% had RA, 29.6% CTD, 9.9% vasculitis and 8.9% other AIDs. The total observation time was 665 patient-years. RTX retention probability at 2 years (95%CI) was similar for RA and CTD 0.65 (0.55 to 0.73), 0.60 (0.47 to 0.72) and lower for vasculitis 0.25 (0.09 to 0.45). Survival curves for RTX retention matched closely (*p* = 0.97) between RA and CTD patients but were lower for patients with vasculitis due to a higher percentage of induced remission. Patients with vasculitis (95%) and CTD (75%) had a higher rate of concomitant glucocorticoid use than RA (60%). Moderate to severe hypogammaglobulinaemia was observed more frequently in patients with vasculitis (35%) than with RA (13%) or CTD (9%) and was associated with an increased risk of presenting a first infectious SAE (HR 2.01, 95% CI 1.04 to 3.91). The incidence rate of SAE was 23.3 SAE/100 patient-years (36% were infectious). When searched, ADAs were observed in 18% of the patients and were detected in 63% of infusions-related SAE. 10 patients died during RTX treatment and up to 12 months after the last RTX infusion, 50% from infection.

**Conclusion:**

RTX retention rates are similar for patients with RA and CTD but lower for those with vasculitis due to more frequent remission. Patients treated with RTX for vasculitis present more SAE and infectious SAE than patients with RA and CTD, potentially due to a higher use of concomitant glucocorticoids and the occurrence of hypogammaglobulinaemia.

**Supplementary Information:**

The online version contains supplementary material available at 10.1186/s13075-023-03076-w.

## Background

Rituximab (RTX) is a chimeric monoclonal antibody targeting CD20, expressed on B cells, inducing a B cell depletion in the peripheral blood. After being used initially to treat non-Hodgkin lymphoma and chronic lymphocytic leukaemia, RTX has proven effective in treating rheumatoid arthritis (RA) and, more recently, in treating ANCA-associated vasculitis (AAV) for induction and maintenance therapy [1–7]. In Switzerland, RTX is licensed to treat severe RA if combined with methotrexate (MTX) after at least one TNF inhibitor failure and, since 2014, to treat severe AAV when cyclophosphamide fails or is contraindicated. For example, RTX may be preferred to treat AAV in young females who plan to have children. The use of RTX to treat other conditions is categorised as off-label, which is associated with coverage issues by the health insurance system. As RTX targets B cells and antibodies production, it is used off-label increasingly often to treat various auto-immune diseases (AID), especially connective tissue diseases (CTD) [8–12]. Several controlled trials studying the efficacy and safety of RTX for AID have been performed with inconsistent results [13–18]. Observational studies investigated the real-life use of RTX to treat AID in national and local registries, but not in Switzerland [19–25].

We aimed to characterise the use of RTX when treating AID in a real-life Swiss setting and compare its use in RA, CTD and vasculitis in terms of efficacy, expressed as drug retention rate, and safety, with a focus on serious adverse events (SAE), especially infectious.

## Methods

### Study design

This was a retrospective study of patients treated with RTX for AID and RA in the Rheumatology Department of the Lausanne University Hospital, a tertiary-care centre. Patients were identified through hospital electronic health records, including pharmacy register and billing data. Medical records were reviewed, and the following information was extracted: demographic data, indication for RTX, initial RTX regimen chosen, number of RTX infusions received, number of cycles of RTX, previous and concurrent immunosuppressive drugs and efficacy and safety outcomes. Comorbidities were recorded, allowing the Charlson Comorbidity Index (CCI) calculation [26]. This study was approved by the Ethics Committee of Canton de Vaud (2018–00385), which waived the requirement to obtain informed consent due to the study’s retrospective design.

### Patients

Patients were included if they were aged > 18 years, treated with RTX for an AID or RA in the Lausanne University Hospital Rheumatology Department, and if RTX was started from 2005 until the end of February 2017, allowing a minimal 2-year duration of follow-up when data extraction began. Patients were excluded if RTX indication was related to onco-haematology (lymphoma, lymphoproliferative disorder) or transplantation medicine.

### Study outcomes

The primary outcome was the RTX retention rate. RTX’s first discontinuation date was defined as the earliest of one of the following: date of death, date of the decision to discontinue RTX, 12 months after the last RTX infusion or date of start of alternative immunosuppressive treatment. The date of discontinuation decision was recorded if the treating physician noted explicitly in the patient’s medical record their decision to discontinue RTX. Patients were censored at the last observation available. Reasons for discontinuation were categorised as inefficacy, remission, and adverse event.

Adverse events (AE) were recorded and defined as SAE when they resulted in death, were life-threatening, required inpatient hospitalisation, prolonged existing hospitalisation, or resulted in a persistent disability. AE was graded as mild, moderate, severe, life-threatening, or lethal according to the Rheumatology Common Toxicity Criteria version 2 [27]. When available, the occurrence of low IgG levels and detection of anti-rituximab antibodies were recorded. Data were cross-referenced with the national death register that includes all deaths occurring in Switzerland, providing dates of death when applicable, which were considered until 12 months after the last RTX infusion.

### Assessment of anti-rituximab levels

Anti-rituximab antibodies levels were assessed on serum samples using ELISA (Lisa-tracker®, Theradiag, France) as previously described [28]. The limit of detection of anti-rituximab antibody levels was 5 ng/mL. These assessments were not performed systematically in all patients but at the request of the treating physician for clinical reasons.

### Statistical analysis

When appropriate, patients’ characteristics were compared by RTX indication with chi-square or Student’s *t*-test. RTX retention rates with 95% CI were calculated through survival analyses. Kaplan–Meier (KM) curves by RTX indication were obtained and compared with log-rank tests. KM curves were right-censored when < 10% of patients were still exposed to RTX. In univariate analysis, Cox regression was used to calculate the hazard ratio (HR) with 95% CI, followed by multivariate analysis on RTX indication adjusted for age, gender, CCI, rheumatoid factor positivity, and conventional disease-modifying anti-rheumatic drugs (csDMARD) treatment. Adverse events were expressed as incidence rate (number of events per 100 patient-years with 95% CI). Time to first SAE was obtained with survival analyses. HR with 95% CI for a first SAE was calculated with Cox regression in a univariate analysis followed by multivariate analysis with adjustment for age, CCI and RTX indication.

## Results

### Patient’s characteristics

Two hundred fourteen patients met the inclusion criteria, of which 11 patients were excluded as they explicitly refused to participate in clinical research. Thus, 203 patients were included in the study, for which RTX was initiated between 2005 and 2017. Mean (standard deviation, SD) and maximal follow-up time was 3.3 (3.2) and 12.8 years, respectively (total observation time: 665 patient-years). Ten patients were lost to follow-up during RTX treatment.

Patients’ mean (SD) age at the time of RTX initiation was 54.7 (16.4) years, and 158 were female (77.8%). The median time between diagnosis and first RTX was 5.5 years (range 0 to 46.5). The median CCI was 1 (range 0 to 10).

Previous treatment before RTX initiation consisted of glucocorticoids (91.0%), csDMARDs (87.7%), anti-TNF agents (55.7%) and other biological agents (20.2%). RTX was combined with glucocorticoids (66.5%), csDMARDs (58.6%) or administered as monotherapy (11.8%).

Indications for RTX consisted of RA (51.7%), CTD (29.6%), vasculitis (9.9%) and other inflammatory conditions (8.9%), detailed in Table 1. Patients’ characteristics by RTX indication are summarised in Table 2.

**Table 1:** Detailed indications for rituximab treatment

Indication for rituximab, *n* (%)	*N* = 203
Rheumatoid arthritis	105 (51.7)
Including ACPA positive	77/105 (73.3)
Including RF positive	79/105 (75.2)
Seronegative rheumatoid arthritis (RF and ACPA negative)	17/105 (16.2)
Connective tissue diseases	
Systemic lupus erythematosus	15 (7.4)
Overlap syndrome (connective tissue disease)	15 (7.4)
Sjögren syndrome	10 (4.9)
Undifferentiated connective tissue disease	8 (3.9)
Dermato-/polymyositis	7 (3.5)
Mixed connective tissue disease (MCTD)	3 (1.5)
Systemic sclerosis	2 (1.0)
Vasculitis	
Microscopic polyangiitis (MPA)	6 (3.0)
Granulomatosis with polyangiitis (GPA)	5 (2.5)
Vasculitis (other)	5 (2.5)
Hepatitis C-associated cryoglobulinemic vasculitis	4 (2.0)
Other	
Ankylosing spondylarthritis	3 (1.5)
Psoriatic arthritis^a^	1 (0.5)
Behçet disease	1 (0.5)
Sarcoidosis	1 (0.5)
Glomerulonephritis	1 (0.5)
Immune thrombocytopenic purpura	1 (0.5)
Autoimmune haemolytic anaemia	1 (0.5)
Antiphospholipid antibody syndrome	1 (0.5)
Myasthenia gravis	1 (0.5)
Interstitial lung disease	1 (0.5)
Scleritis	1 (0.5)
Autoimmune hepatitis	1 (0.5)
Primary biliary cholangitis-related arthritis	1 (0.5)
Calcium pyrophosphate dihydrate crystal-related arthritis^a^	1 (0.5)
Hereditary myopathy^a^	1 (0.5)
Motor neurone disease^a^	1 (0.5)

^a^Incorrect initial diagnosis

**Table 2:** Patients’ characteristics by rituximab indication

	**RA *n* = 105**	**CTD *n* = 60**	**Vasculitis *n* = 20**	**Other *n* = 18**	**Comparison between groups**
Age at time of first RTX, years, mean (SD)	57.8 (13.9)	48.0 (14.3)	57.4 (14.1)	56.2 (13.7)	*p* = 0.0003
Female, *n* (%)	82 (78.1)	55 (91.7)	10 (50.0)	11 (61.1)	*p* < 0.0001
Time between diagnosis and first RTX, years, median [range]	7.3 [0.3, 46.5]	5.0 [0.1, 38.7]	0.4 [0.02, 10.8]	2.4 [0.4, 31.1]	*p* = 0.0001
Previous treatments, *n* (%)					
Glucocorticoids	87/91 (95.6)	51/59 (86.4)	19 (95.0)	14 (77.8)	*p* = 0.045
csDMARD (any)	103 (98.1)	55 (91.7)	6 (30.0)	14 (77.8)	*p* < 0.0001
Cyclophosphamide	0	4 (6.7)	11 (55.0)	2 (11.1)	*p* < 0.0001
Anti-TNF agent	88/103 (85.4)	18 (30.0)	0	6 (33.3)	*p* < 0.0001
Other biological agents	30 (28.6)	6 (10.0)	0	5 (27.8)	*p* = 0.003
Initial RTX regimen					*p* < 0.0001
1000 mg/15 days (× 2)	102 (97.1)	50 (83.3)	8 (40.0)	14 (77.8)	
375 mg/m^2^/week (× 4)	0	5 (8.3)	11 (55.0)	4 (22.2)	
other	3 (2.9)	5 (8.3)	1 (5.0)	0	
Concomitant treatments, *n* (%)					
Glucocorticoids	56/94 (59.6)	41/55 (74.6)	19 (95.0)	7/16 (43.8)	*p* = 0.002
csDMARD (any)	71 (67.6)	39 (65.0)	3 (15.0)	6 (33.3)	*p* < 0.0001
Methotrexate	46/98 (46.9)	14/59 (23.7)	1 (5.0)	2/16 (12.5)	*p* < 0.0001
None	12 (11.4)	5 (8.3)	1 (5.0)	6 (33.3)	*p* = 0.02
IgG levels before RTX initiation, g/l, mean (SD)	11.3 (3.9) *n* = 94	13.7 (7.8) *n* = 52	9.6 (4.4) *n* = 17	9.6 (2.7) *n* = 14	*p* = 0.05
Hypogammaglobulinaemia before RTX initiation, *n* (%)					*p* = 0.002
None	84/94 (89.4)	44/52 (84.6)	12/17 (70.6)	12/14 (85.7)	
Mild (5–6.9 g/l)	10/94 (10.6)	7/52 (13.5)	2/17 (11.8)	2/14 (14.3)	
Moderate to severe (< 5 g/l)	0	1/52 (1.9)	3/17 (17.7)	0	

*RA* Rheumatoid arthritis, *CTD* Connective tissue disease, *RTX* Rituximab

RTX prescriptions were off-label for 43% of them, according to the Swiss drug agency-approved indications list. 60% of RTX prescriptions for RA did not totally fulfil the approved label (previous anti-TNF treatment and concomitant treatment with MTX).

As individual RTX infusion doses were not recorded, the cumulative dose of RTX could not be calculated. We observed various treatment regimens, but most patients were treated with two RTX infusions twice a year. We provide the number of RTX cycles and infusions per year and by indication for patients treated for one year or more in Supplementary Table 1.

### Rituximab retention rate

**Fig. 1:**
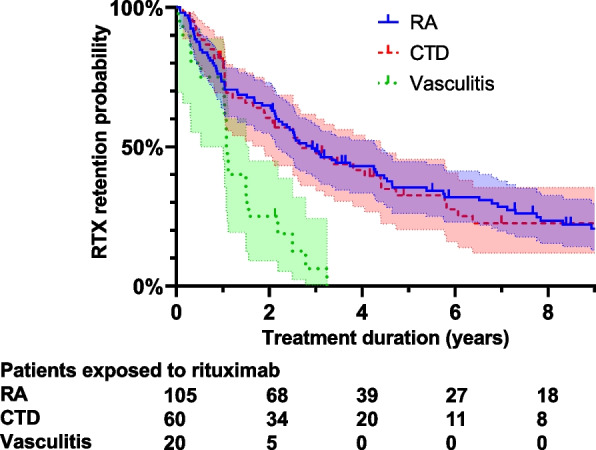
Rituximab retention rate. Kaplan–Meier curve showing the retention probability of rituximab treatment by indication with 95% CI

RTX retention probability was 0.73 (95% CI 0.67, 0.79), 0.55 (95% CI 0.48, 0.61) and 0.28 (95% CI 0.22, 0.35) at 1 year, 2 years and 5 years after treatment initiation, respectively. RTX retention probability for patients with RA, CTD and vasculitis at 2 years was 0.65 (95% CI 0.55, 0.73), 0.60 (95% CI 0.47, 0.72) and 0.25 (95% CI 0.09, 0.45) and at 5 years was 0.35 (95% CI 0.26, 0.45), 0.33 (95% CI 0.20, 0.45) and 0, respectively. After restricting the analysis to the 12 AAV, RTX retention probability at 2 years was 0.42 (95% CI 0.15, 0.67). Nineteen per cent of patients were still under RTX treatment after 8 years. The RTX retention rate of patients was similar between patients with RA and CTD (*p* = 0.97) (Fig. 1) but differed significantly compared to vasculitis (*p* < 0.0001). The RTX first discontinuation date used for the survival analysis was the date of the decision to discontinue RTX by the treating physician (35%), the start of alternative treatment (34%), 12 months after the last RTX infusion (26%) and the date of death (4%).

RTX was discontinued in 161 patients, and reasons for discontinuation were known for 138 of them: inefficacy (51.5%), AE (26.1%) or remission (22.4%). Reasons for drug discontinuation by indications are detailed in Table 3. RTX treatment was more likely to be discontinued due to remission for patients with vasculitis and due to inefficacy for RA and CTD. 12 of 31 (39%) patients presented a disease relapse after RTX discontinuation for remission (RA 4/7 57%, CTD 2/9 22%, vasculitis 5/13 39%) and RTX treatment was resumed in 10 of these patients. A relapse was observed after a mean time of 673 days (RA 484 days, CTD 360 days, vasculitis 693 days). RTX was discontinued in 7/12 patients with AAV after less than two years of treatment. RTX was resumed due to a relapse of AAV in 3 of them. For patients with “other inflammatory diseases”, RTX was discontinued essentially due to inefficiency (75%) and adverse events (13%). In 2 of them, RTX was discontinued for remission. They were suffering from idiopathic thrombocytopenic purpura and autoimmune haemolytic anaemia.

**Table 3:** Reasons for rituximab discontinuation by indication

Rituximab discontinuation	RA *n* = 68	CTD *n* = 35	Vasculitis *n* = 19	Other *n* = 16	All *n* = 138
Remission,* n* (%)	7 (10.3)	9 (25.7)	13 (68.4)	2 (12.5)	31 (22.5)
Inefficacy, *n* (%)	36 (52.9)	19 (54.3)	4 (21.1)	12 (75.0)	71 (51.5)
Adverse event, *n* (%)	25 (36.8)	7 (20.0)	2 (10.5)	2 (12.5)	36 (26.1)

Comparison between groups:* p* < 0.0001. 23 missing values. *RA* Rheumatoid arthritis, *CTD* Connective tissue disease

In univariate analysis, RTX retention probability was increased when patients were positive for rheumatoid factor (HR 0.62 [95% CI 0.45, 0.86]), had been previously (HR 0.62 [95% CI 0.39, 0.97]) or concomitantly (HR 0.61 [95% CI 0.44, 0.84]) treated by any csDMARD, when full B cells depletion was observed (HR 0.44 [95% CI 0.20, 0.96]) and with time elapsed (years) between diagnosis and first RTX (HR 0.976 [95% CI 0.956, 0.997], *p* = 0.02).

In univariate and multivariate analyses, after adjusting for gender, age at RTX initiation, rheumatoid factor positivity, previous and concurrent use of csDMARD, RTX retention probability was similar for patients with CTD compared to RA and decreased for patients with vasculitis and other inflammatory conditions, see Table 4.

**Table 4:** Rituximab first discontinuation hazards ratio by indication and other factors of interest, multivariate Cox regression analysis

Rituximab first discontinuation	Adjusted Hazard ratio^a^	95% CI	*P*
Rituximab indication			*p* = 0.004
RA	Reference		
CTD	0.87	0.57, 1.33	
Vasculitis	2.45	1.13, 5.30	
Other inflammatory conditions	2.42	1.25, 4.68	
Age at first RTX	1.00	0.99, 1.01	*p* = 0.96
Gender: male	0.98	0.66, 1.45	*p* = 0.91
Charlson comorbidity index	1.10	0.98, 1.24	*p* = 0.11
RF positivity	0.61	0.42, 0.90	*p* = 0.01
Previous treatment with csDMARD (any)	1.26	0.63, 2.53	*p* = 0.51
Concomitant treatment with csDMARD (any)	0.74	0.52, 1.06	*p* = 0.10

*RA* Rheumatoid arthritis, *CTD* Connective tissue disease, *RTX* Rituximab, *RF* Rheumatoid factor

^a^Factors are adjusted for each other

### Safety

One hundred fifty-five SAEs were reported and occurred in 73/203 (36.0%) patients, of which 56 (36.1%) were serious infectious events (SIEs), which occurred in 37/203 (18.2%) patients. The severity of SAEs was mild in 9.7%, moderate in 46.5%, severe in 25.2%, life-threatening in 6.5% and lethal in 12.3%. Fifty-four SAEs (36%) occurring in 36 patients leaded to a RTX discontinuation and 83% of SAEs resolved. 10 patients died during RTX treatment and up to 12 months after RTX discontinuation. Their characteristics are detailed in Table 5. Incidence rates of SAEs and SIEs were significantly higher for patients with vasculitis than patients with RA and CTD. Still, incidence rates of infusion-related reactions and deaths did not significantly differ between indications for RTX, although they were numerically higher for patients with vasculitis (Table 6).

**Table 5:** Characteristics of patients deceased during rituximab treatment and up to 12 months after treatment discontinuation

Rituximab indication	Gender	Charlson comorbidity index	Age at first rituximab, years	Treatment duration, years	Time from last rituximab to death, days	Cause of death
RA	F	5	48	2.8	63	Pulmonary aspergillosis
RA	M	0	75	9.7	62	*Pneumocystis jirovecii* pneumonia
RA	M	2	81	1.2	179	Sepsis secondary to pulmonary aspergillosis and haemorrhagic shock secondary to bleeding duodenal ulcer
RA	F	9	61	0.04	189	Lung cancer
SLE	F	5	34	1.4	41	Miliary tuberculosis
MCTD	F	1	49	6.2	60	Progressive multifocal leukoencephalopathy
GPA	M	4	63	2.9	221	Unknown
Hepatitis C-associated cryoglobulinemic vasculitis	F	6	85	0.02	9	Hepatic and renal failure
Hepatitis C-associated cryoglobulinemic vasculitis	M	5	77	6.4	128	Sepsis due to *Escherichia coli*
Interstitial lung disease	M	4	62	0.04	152	Unknown

*RA* Rheumatoid arthritis, *SLE* Systemic lupus erythematous, *MCTD* Mixed connective tissue disease, *GPA* Granulomatosis with polyangiitis

**Table 6:** Incidence rate of selected adverse events by rituximab indication

	**Incidence rate [95% CI]**				
	**Total**	**RA**	**CTD**	**Vasculitis**	**Other**
SAE/100 patient-years	23.3 [19.8, 27.3]	21.8 [17.5, 26.8]	15.3 [10.5, 21.6]	106.9 [71.1, 154.5]	28.6 [9.3, 66.7]
SIE/100 patient-years	8.4 [6.4, 10.9]	8.7 [6.1, 12.1]	4.8 [2.3, 8.8]	34.4 [15.7, 65.2]	5.7 [0.1, 31.8]
IRR/100 patient-years	4.2 [2.8, 6.1]	3.3 [1.9, 5.7]	2.9 [1.1, 6.2]	15.3 [4.2, 39.1]	22.9 [6.2, 58.5]
Malignancies/100 patient-years	0.8 [0.2, 1.8]	0.7 [0.1, 2.1]	1.0 [0.1, 3.5]	0	0
Deaths/100 patient-years	1.50 [0.81, 2.79]	0.97 [0.36, 2.58]	0.96 [0.24, 3.83]	11.5 [3.70, 35.5]	5.71 [0.80, 40.6]
Observation time, patient-years	665.2	412.5	209.0	26.2	17.5

*RA* Rheumatoid arthritis, *CTD* Connective tissue disease, *SAE* Serious adverse event, *SIE* Serious infectious event, *IRR* Infusion-related reaction

Hypogammaglobulinaemia was observed in 58/171 (33.9%) patients and was categorized as mild (5–6.9 g/l) in 21.0%, moderate (3–4.9 g/l) in 10.5% or severe (< 3 g/l) in 2.3%. Moderate to severe hypogammaglobulinaemia occurred in 12.6%, 9.3% and 35.3% of patients with RA, CTD and vasculitis, respectively (*p* = 0.04). Occurrence of hypogammaglobulinaemia was associated with age at RTX initiation (OR 1.05 [95% CI 1.03, 1.08]), CCI (OR 1.21 [95% CI 1.002, 1.47]), concomitant use of glucocorticoids (OR 2.66 [95% CI 1.24, 5.74]) and inversely associated with IgG levels before RTX initiation (OR 0.71 [95% CI 0.62, 0.82]).

Anti-drug antibodies (ADA) were observed in 11/61 (18.0%) of the patients. Median time between assessment of ADAs and the last rituximab infusion was 168 days (IQR 55). Detection of ADAs was positively associated with anti-SSA positivity (OR 4.95 [95% CI 1.16, 21.1]), concomitant use of hydroxychloroquine (OR 4.88 [95% CI 1.17, 20.4]) and diagnosis of CTD (OR 6.5 [95% CI 1.19, 35.6]) and negatively associated with rheumatoid factor (RF) positivity (OR 0.17 [95% CI 0.04, 0.79]) and previous treatment with MTX (OR 0.16 [95% CI 0.04, 0.65]). Among 56 SAEs where ADA were searched for, ADA were detected more often (*p* = 0.03) for infusion-related reactions (62.5%) than infections (9.5%) or death (16.7%). Infusion-related reactions (*n* = 28) were graded as mild (46%), moderate (32%), severe (18%), life-threatening (4%) and 46% of them were categorized as SAE.

Patients not previously treated with methotrexate, older than 60, or in whom ADAs were detected or B cells depletion was not obtained, had an increased risk of presenting a first SAE (Table 7) in multivariate analysis. Patients with vasculitis had a higher risk of presenting a first SIE (HR 2.76 [95% CI 1.09, 6.99]) than patients with RA. The risk to present a first SIE over time was associated with moderate to severe hypogammaglobulinaemia but not directly associated with use of glucocorticoids (Table 8).

**Table 7:** Risk factors associated with a first serious adverse event during rituximab treatment

	**Unadjusted Hazard ratio**	**95% CI**	Adjusted Hazard ratio^a^	**95% CI**
Age at first RTX				
< 40 years	Reference		Reference	
40 to 60 years	1.06	0.42, 2.68	1.04	0.41, 2.69
> 60 years	3.86	1.65, 9.03	3.21	1.29, 7.95
Charlson comorbidity index				
0	Reference		Reference	
1	0.64	0.28, 1.47	0.66	0.28, 1.59
2	0.68	0.24, 1.87	0.72	0.25, 2.05
≥ 3	2.60	1.12, 6.02	1.93	0.79, 4.68
Anti-drug antibodies identification	2.73	1.12, 6.61	3.05	1.01, 9.18
Previous treatment with MTX	0.49	0.30, 0.79	0.47	0.24, 0.90
B cells depletion (≤ 5 cell/mm^3^)	0.31	0.12, 0.78	0.27	0.09, 0.82

^a^Adjusted for: age at first RTX (categorised), Charlson comorbidity index (categorised), RTX indication (RA, CTD, vasculitis, other)

*MTX* methotrexate, *RTX* rituximab

**Table 8:** Risk factors associated with a first serious infectious adverse event during rituximab treatment

	**Unadjusted Hazard ratio**	**95% CI**	**Adjusted Hazard ratio**^**a**^	**95% CI**
Gender: male	2.03	1.02, 4.07	1.52	0.71, 3.26
Age at first RTX				
< 40 years	Reference		Reference	
40 to 60 years	1.36	0.38, 4.87	1.16	0.32, 4.26
> 60 years	3.02	0.90, 10.1	1.84	0.51, 6.60
Charlson comorbidity index				
0	Reference		Reference	
1	0.33	0.13, 0.87	0.38	0.13, 1.06
2	0.41	0.11, 1.45	0.42	0.11, 1.57
≥ 3	1.71	0.65, 4.52	1.21	0.41, 3.59
Concurrent use of glucocorticoids	0.99	0.49, 1.99	1.06	0.48, 2.33
Hypogammaglobulinaemia				
None	Reference		Reference	
Mild (5–6.9 g/l)	1.29	0.53, 3.11	1.44	0.55, 3.76
Moderate to severe (< 5 g/l)	3.13	1.47, 6.70	2.54	1.03, 6.28
RTX indication				
RA	Reference		Reference	
CTD	0.49	0.20, 1.20	0.87	0.32, 2.35
Vasculitis	2.76	1.09, 6.99	1.79	0.62, 5.14

^a^Adjusted for: age at first RTX (categorised), Charlson comorbidity index (categorised), RTX indication (RA, CTD, vasculitis, other)

*RTX* rituximab, *RA* Rheumatoid arthritis, *CTD* Connective tissue disease

## Discussion

To our knowledge, this is the first study comparing rituximab retention when treating RA versus other AID patients, in particular CTDs and vasculitis, in a real-life setting.

RTX retention rate was the lowest for patients with vasculitis, in which it was generally discontinued due to remission, but was similar between patients with RA and CTD. For these patients, RTX was discontinued most often due to inefficacy. These results remained unchanged after adjustment for potential confounders such as age, gender, comorbidities, and csDMARDs treatment. RTX was frequently used off-label (43%) to treat AID, especially CTD. When treating RA, prescription most often only partially fulfilled the approved label, for example, when RTX was not combined with methotrexate or used as a first-line treatment.

The RTX retention rate for patients with RA of 65% [95% CI 55%, 73%] at 2 years and 35% [95% CI 26%, 45%] at 5 years is in line with the British Society for Rheumatology Biologics Register for RA data (RTX retention at 2 years: 64.9%) [29], the French Autoimmunity and Rituximab registry (AIR) data (RTX retention at 2 years: 67.6%) [30] and the Swedish Rheumatology Register (RTX retention at 2 years: 63.5% and 5 years: 24.8%) [31], while reasons for discontinuation had similar percentages. Although randomised trials have failed so far to provide definite evidence supporting RTX to treat CTD such as SLE [17, 18] or SS [13, 14], RTX is routinely used to treat these conditions, especially in patients with refractory diseases [9, 10, 12]. Indeed, we observed that CTD accounted for 30% of RTX prescriptions. The RTX retention survival curve closely matched that of RA, suggesting a similar efficacy profile to RA, as reasons for discontinuation of RTX were identical. Subgroups analyses for specific conditions among CTDs were not performed due to the limited number of participants. RTX retention rate for vasculitis differed drastically compared to CTD or RA. In most cases, RTX use started soon after diagnosis and was discontinued due to remission for 68% of the patients. These results align with those of randomised trials that demonstrated RTX efficacy for induction and maintenance therapy in AAV [2–7]. After multivariate analysis using the Cox proportional hazards model with adjustment for potential confounders, we found similar HR of RTX discontinuation by indication as with univariate analysis, underlining the robustness of our results.

Our safety data showed a higher incidence rate of SAE for RA compared to pooled safety data of the clinical development programme of RTX (21.8 [17.5 to 26.8] versus 14.4 [13.7 to 15.1] SAEs/100 patient-years) [32] and Greek post-marketing surveillance data (6.8 SAEs/100 patient-years) [33]. Patients included in clinical trials are generally less likely to present AE than patients treated in real-life settings. RTX is usually used in Switzerland as a third line (or higher) therapy for RA, therefore selecting patients with a refractory disease, which could explain the difference with Greek data, where the treatment algorithm for RA may differ.

German (GRAID), French (AIR) and Spanish (BIOGEAS) registries, including patients treated with RTX for AID, reported an incidence rate of SIEs of 5.3, 3.8 and 6.4 (SLE only) SIEs/100 patient-years respectively, which are close to our observations (4.8 SIEs/100 patient-years for CTD) [11, 34, 35]. Our results are also close to the results of a Swiss retrospective study (5.9 and 24.9 SIEs/100 patient-years for RA and AID, respectively) [36]. A significant proportion of patients presented reduced IgG levels (< 7 g/l) during treatment with RTX (34%), which is higher than another French long-term longitudinal study (17%) that included 134 patients with RA [37]. Low IgG levels were associated with an increased risk of presenting a first SIE, consistent with other studies that also identified low IgG levels as a risk factor for infectious events [36–39]. Interestingly, concurrent use of glucocorticoids was not directly associated with an increased risk of SAE or SIE in crude and adjusted analysis but was positively associated with the occurrence of hypogammaglobulinaemia. We observed the development of ADAs in 18% of patients when searched for, which is in line with other studies [40, 41] and their presence was associated with an increased risk of infusion-related reaction (IRR). Thus, in clinical practice, the assessment of ADAs may be considered in the case of IRR as their presence may lead to a change of anti-CD20 agent.

Patients with vasculitis treated with RTX presented a significantly higher incidence rate of SAEs, SIEs and mortality, which could be related to a more frequent concomitant use of glucocorticoids, a higher incidence of hypogammaglobulinaemia and to the disease’s natural course, which is usually more severe than RA or CTDs. Ten patients, the most suffering from many comorbidities, died during RTX treatment and up to 12 months after the last RTX infusion (1.5 deaths/100 patient-years), half of whom from an infection, which was due to an opportunistic pathogen for 4 patients.

Our data argue for taking all possible measures to reduce the risk of infections in patients treated with RTX. For example, the regular measurement of IgG levels may allow early detection of hypogammaglobulinaemia and timely introduction of intravenous immunoglobulin substitution. Ideally before RTX initiation, immunisations should be systematically offered to our patients according to national guidelines. Protocols allowing the use of a reduced dose of glucocorticoids, such as in the PEXIVAS trial, should be followed [42].

This study is limited by its retrospective design, which can impact reporting of AE, IgG levels and the development of ADAs during the follow-up. As ADAs were not assessed systematically in all patients, it may introduce bias in the interpretation of the association of their presence with specific clinical conditions. Detailed dosing of concomitant glucocorticoids was not recorded, which may impede the analysis of their association with infectious risk. This study also included only patients with conditions treated by our Rheumatology Department and did not include patients suffering from neurologic, dermatologic, or purely hematologic diseases. However, as RTX administration dates were extracted from the electronic health records, the RTX retention rate, the primary outcome, was unlikely to be affected by the study design. We also improved the mortality estimate accuracy by cross-linking our data with the Swiss death register. Although the number of included patients was relatively small, preventing subgroup analyses for each condition, the length of follow-up and the low percentage of lost to follow-up are strengths of this study.

This study reflects real-life practice from 2005 to 2019, before the COVID-19 pandemic. The results would probably be different if this study had been repeated today. Indeed, as RTX was associated with an increased risk of severe COVID-19, COVID-19-related hospital admission and death, there is a tendency to use RTX only if no alternative treatment with a shorter half-life is available across indications [43]. In addition, as new treatments are available for RA (JAK inhibitors) and SLE (belimumab, anifrolumab), RTX is used more frequently as a third (or higher) line of treatment than previously. On the other hand, when treating AAV, RTX is usually preferred over alternative treatments (cyclophosphamide) and is now used routinely for induction and maintenance therapy due to new guidelines and recent trials, leading to a more prolonged RTX patient exposure.

## Conclusions

RTX retention rate was similar between patients with RA and CTD, suggesting a similar efficacy and was most often discontinued due to a lack of efficacy. The RTX retention rate was lower for patients with vasculitis, for which RTX was generally discontinued because of remission. Patients with vasculitis presented more SAE, SIE and higher mortality than patients with RA or CTD. Occurrence of SIEs was associated with moderate to severe hypogammaglobulinaemia and a more frequent use of concomitant glucocorticoids.

## Supplementary Information

**Additional file 1: Supplementary Table 1. **Number of rituximab cycles and infusions per year by rituximab indication for patients treated ≥ 1 year.

## Data Availability

The datasets used and/or analysed during the current study are available from the corresponding author on reasonable request.

## Abbreviations

AAV: ANCA-associated vasculitis
ADA: Anti-drug antibodies
AE: Adverse events
AID: Auto-immune diseases
AIR: French Autoimmunity and Rituximab registry
CCI: Charlson Comorbidity Index
CTD: Connective tissue diseases
csDMARD: Conventional disease-modifying anti-rheumatic drugs
HR: Hazard ratio
IRR: Infusion-related reaction
KM: Kaplan-Meier
MTX: Methotrexate
RA: Rheumatoid arthritis
RF: Rheumatoid factor
RTX: Rituximab
SAE: Serious adverse events
SIE: Serious infectious events
