# Supplementary material for: Real-life drug retention rate and safety of rituximab when treating rheumatic diseases: a single-centre Swiss retrospective cohort study
Source: Arthritis Res Ther. 2023 Jun 1;25:91. doi: 10.1186/s13075-023-03076-w (PMC10233194; doi:10.1186/s13075-023-03076-w)
Supplement: Supplementary file 1 — Additional file 1: Supplementary Table 1. Number of rituximab cycles and infusions per year by rituximab indication for patients treated ≥ 1 year. [file 13075_2023_3076_MOESM1_ESM.docx]

**Supplementary Table 1 Number of rituximab cycles and infusions per year by rituximab indication for patients treated ≥ 1 year.**

RA: rheumatoid arthritis, CTD: connective tissue disease, RTX: rituximab

| **Patients treated with RTX ≥ 1 year and with available data** | **RA n=72** | **CTD  n=40** | **Vasculitis n=10** | **All n=122** |
| --- | --- | --- | --- | --- |
| Duration of treatment (years)* mean (SD) median [range] | 5.5 (3.5) 4.6 [1.0-12.4] | 4.9 (3.3) 4.1 [1.0-11.9] | 3.8 (3.0) 2.2 [1.6-10.4] | 5.2 (3.4) 4.3 [1.0-12.4] |
| Number of cycles of RTX mean (SD) median [range] | 9.7 (7.1) 6.5 [3-34] | 8.5 (5.2) 7.0 [2-21] | 5.3 (2.9) 4.5 [3-11] | 8.9 (6.4) 6.0 [2-34] |
| Number of RTX infusions mean (SD) median [range] | 18.3 (13.5) 12.5 [3-66] | 16.0 (10.2) 13.0 [4-45] | 7.8 (3.3) 7.5 [3-13] | 16.7 (12.2) 12.0 [3-66] |
| Number of cycles of RTX per year  mean (SD) median [range] | 1.86 (0.56) 1.95 [0.33-2.97] | 1.95 (0.58) 2.02 [0.28-2.95] | 1.71 (0.63) 1.71 [0.47-2.57] | 1.87 (0.57) 1.97 [0.28-2.97] |
| Number of RTX infusions per year mean (SD) median [range] | 3.51 (1.14) 3.89 [0.55-5.93] | 3.64 (1.14) 3.56 [0.83-5.91] | 2.52 (0.94) 2.37 [1.25-3.77] | 3.47 (1.16) 3.53 [0.55-5.93] |
| Number of RTX infusions per cycle mean (SD) median [range] | 1.88 (0.15) 1.94 [1.4-2] | 1.91 (0.33) 2 [1.17-3] | 1.65 (0.91) 1.25 [1-4] | 1.87 (0.34) 1.94 [1-4] |

**Duration of treatment: time between the first RTX infusion and the last known infusion at the time of data collection.*
